# Interactions of a Forced Vibrating Membrane with a Cylindrical Acoustic Cavity

**DOI:** 10.3390/s25237117

**Published:** 2025-11-21

**Authors:** Manuel Gascón-Pérez

**Affiliations:** Escuela Técnica Superior de Ingeniería Aeronáutica y del Espacio, Universidad Politécnica de Madrid, Pza. Cardenal Cisneros s/n, 28040 Madrid, Spain; manuel.gascon@upm.es; Tel.: +34-91-067-5737

**Keywords:** top membrane of cylindrical cavity, fluid–structure interaction, natural frequencies, frequency response forced vibrating membrane, compressibility effects, fluid velocity potential

## Abstract

Acoustic cavities play a role in many technological applications in civil, naval, and aerospace engineering. This study examines the vibroacoustic performance of a forced oscillating top membrane of a cylindrical container fully filled with a compressible and nonviscous fluid. For the case of harmonic motion and using Helmholtz’s equation, the velocity potential is deduced, and the acoustic pressure is obtained using Bernoulli’s linearized equation. Taking into account the dynamic equation for the membrane with the interacting fluid with the different terms expanded in a modal series and after an integration procedure over the membrane surface, a simple analytical quadratic equation is deduced, and the coupled natural frequencies of the membrane are obtained. For the case of forced vibrations, a transfer function is obtained for calculating the frequency spectrum response of the fluid–membrane interacting system. In particular, the membrane deformation spectrum and the acoustic cavity pressure spectrum are obtained for different location points. Moreover, the spectrum of the mean quadratic values of the membrane deflexion and acoustic pressure are deduced, along with its variation with different parameters such as drum height, membrane radius, fluid density, load position, sound speed, and membrane tension. The variation in sensitivity with frequency and other different parameters is also analysed. The results are contrasted with those obtained by other authors to validate the present work.

## 1. Introduction

The vibration of an elastic cover over an acoustic cavity is a fluid–structure interaction problem encountered in many engineering applications, such as in aerospace, civil, or naval fields. It is encountered in aircraft and ship cabins, civil rooms, and sonar cavities.

This structure-borne noise can affect the comfort of passengers and operators in the cabins of aircraft, ships, or land vehicles, and is modelled in a simplified way with a vibrating elastic wall of an acoustic cavity. An appropriate analysis of the interaction between the fluid and structure of the acoustic cavity is of critical interest to control the sound in these cabins and acoustic containers and has been studied for a long time. The mental and physiological states of the pilots and passengers can be seriously affected when the acoustic pressure level increases, so special care must be considered regarding acoustic behaviour when designing these devices in order to reduce the acoustic noise.

There are also components found in MEMs (micro electromechanical systems) that have similarities with those of acoustic cavities. For the case of micropumps, Laser et al. [1], Pan et al. [2], and Shabani et al. [3] analysed the free frequencies for the purpose of controlling the power consumption and efficiency. For the case of pressure sensors, see Defay et al. [4], where the elastic structural member is excited at resonant conditions (natural frequencies).

Many engineering problems regarding an acoustic cavity are modelled simply, considering cylindrical or rectangular geometries. Early in 1963, Dowell and Voss [5] studied the oscillations of a panel in an acoustic rectangular cavity for a compressible fluid, expanding in a Fourier series the expressions for panel deformation and the acoustic pressure. Bauer [6] considered the vibration of an elastic membrane of a cylindrical cavity employing a variational method and considering the fluid incompressible. The forced vibrations of the top elastic member of a rectangular cavity are analysed in [7,8] by Pretlove.

Recently, researchers have been more concerned with the active control of cabin acoustics (see Pan et al. [9]), sound radiation structural control (see Qiu [10]), and control of sound transmission (see Kim et al. [11]). Sometimes, a particular approach is employed using a modal coupling analysis between the vacuum modes of the structure and those that correspond to an acoustical rigid cavity, but this is limited to the case of light coupling and cannot be used for cases of small container height, dense fluid, or thin plates. Furthermore, the continuity condition on the fluid–structure boundary is not fulfilled.

FEM and BEM are used for more complex structural geometries. Zienkiewicz and Newton [12] modelled the interaction between a structure and a compressible fluid using the finite element technique. For cases of more complex geometries, Sestieri et al. [13] made an analysis of the acoustic–structural coupling using Kirchoff–Helmholtz`s integral equation. In [14], Xie et al. made use of a variational method for an elastic plate in an irregular cavity considering different boundary edge conditions and wall impedances for the coupled system.

The hydro-elastic vibrations of a membrane of a rectangular container with an incompressible and inviscid fluid are analysed in [15] by Gascón-Pérez. In [16], they considered a vibrating elastic plate inside a rectangular cavity filled with a compressible fluid. Furthermore, in [17], the same author analysed the radiation pattern of an elastic panel fully immersed in an acoustic medium.

The acoustic behaviour of a harmonic forced vibrating membrane of a cylindrical container are analysed in this paper, while considering the fluid compressible and non-viscous. The wave equation is used for the calculus of fluid potential and the pressure field is deduced from Bernoulli`s equation. Expanding the dynamic equation of the membrane in the series of associated modes, and integrating the different terms taking into account their orthogonality, the membrane free frequencies of vibration and the system frequency spectrum response for the case of forced vibration are obtained in a simple way, considering analytical expressions. Different authors have studied a similar problem for an acoustic cavity backed with an elastic member, and in many cases, for a rectangular geometry. They usually analyse the sound pressure level spectrum of a fluid point inside the cavity and the deformation spectrum of a particular point of the elastic member, but for the present study, the mean quadratic sound pressure level over the membrane is analysed, along with the mean quadratic membrane deformation and the mean quadratic sensitivity level; see Jig T. Du et al. [18]. For validation of the method, different results are compared with those obtained by the analyses of other authors.

## 2. Problem Formulation

The vibrating response of the membrane of a cylindrical drum is modelled by the following equation; see Wang [19]:

(1) $$T\Delta \eta (r,\theta ,t)-\rho_{m}t_{h}\frac{\partial^{2}\eta }{\partial t^{2}}=p(r,\theta ,H,t)+\delta q_{0}(r,\theta ,t)$$

where T is the membrane tension, $t_{h}$ the plate thickness, $\rho_{m}$ the material density of the membrane, and η the membrane deformation in the normal direction.

The elastic membrane is in contact with the fluid and p is the acoustic pressure acting over the membrane.

$\delta q_{0}(r,\theta ,t)$ is the harmonic loading force per unit area expressed as a Dirac-delta function located at position $r_{0},\theta_{0}$ and associated with a force $q_{0}$ of 1 N, so that

(2) $$\delta q_{0}(r,\theta ,t)=\delta {\tilde{q}}_{0}(r,\theta )\cdot e^{i\omega t}$$

and

(3) $$q_{0}=\iint_{S_{m}}\delta {\tilde{q}}_{0}\left(r-r_{0},\theta -\theta_{0}\right)\;rdr\;d\theta$$

**Figure 1:**
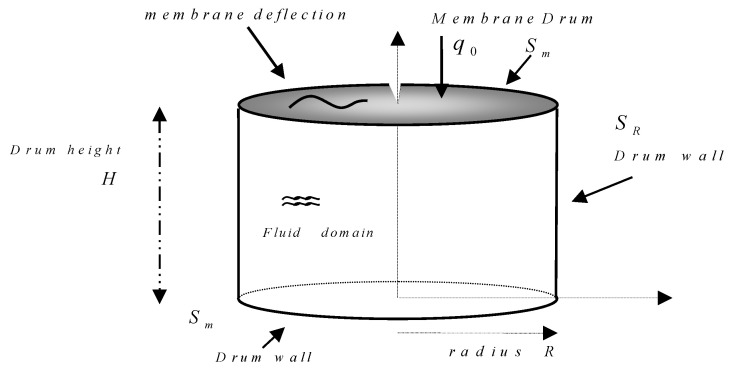
Scheme of a membrane drum showing the different geometrical parameters.

For a membrane located at a position z=H, and with the radius of the membrane R, and drum total height H, see Figure 1.

For a harmonic permanent motion for both the fluid and the membrane, the fluid pressure and deformation have the expressions

(4) $$\eta (r,\theta ,t)=\tilde{\eta }(r,\theta )\cdot e^{i\omega t}\mathrm{and}\;p(r,\theta ,z,t)=\tilde{p}(r,\theta ,z)\cdot e^{i\omega t}$$

The membrane deflection, in contact with the fluid, is expressed as a series expansion of the membrane modes in vacuum:

(5) $$\eta (r,\theta ,t)=\sum_{m,n}\eta_{m}^{n}(r,\theta )\cdot \;{\bar{d}}_{m}^{n}\cdot e^{i\omega_{m}^{n}t}\;$$

That corresponds to the solution of the equation of the vibrating membrane in a vacuum for harmonic motion, i.e.,

(6) $$\Delta \tilde{\eta }(r,\theta )+\beta^{2}\;\tilde{\eta }(r,\theta )=0$$

where ${\bar{d}}_{m}^{n}$ are the loading coefficients associated with the modes that contribute to the total deflection of the circular membrane covered by the fluid.

The function modes $\eta_{m}^{n}\left(r,\theta \right)$ are the solutions of Equation (6), $\Delta \eta_{m}^{n}(r,\theta )+{\beta_{m}^{n}}^{2}\;\eta_{m}^{n}=0$, and for a circular membrane clamped along its edge, they have this expression; see Wang [19]:

(7) $$\eta_{m}^{n}(r,\theta )=J_{m}\left(\beta_{m}^{n}r\right)\cos(m\theta )$$

where $J_{m}$ is the Bessel function of the first kind of the $m^{\mathrm{th}}$ order, and $\beta_{m}^{n}$ is a parameter that depends on different properties of the membrane and gives the frequencies of the membrane in a vacuum, $\beta_{m}^{n}=\omega_{m}^{n}\sqrt{\frac{\rho_{m}t_{h}}{T}}$, and is calculated from the roots of equation $J_{m}\left(\beta_{m}^{n}R\right)=0$. The indexes m,n represent the number of nodal diameters and circles, respectively.

The concentrated load is expressed in a series of the deflection modes in a vacuum:

(8) $$\delta {\tilde{q}}_{0}(r,\theta )=\sum_{m}\sum_{n}{\bar{q}}_{m}^{n}\cdot \eta_{m}^{n}(r,\theta )$$

where the weight coefficients are expressed as

(9) $${\bar{q}}_{m}^{n}=\frac{2}{\pi R^{2}\Lambda_{\beta m}^{n}}\iint_{S_{m}}\delta {\tilde{q}}_{0}(r-r_{0},\theta -\theta_{0})\cdot \eta_{m}^{n}(r,\theta )rdrd\theta =\frac{2}{\pi R^{2}\Lambda_{\beta m}^{n}}q_{0}\cdot \eta_{m}^{n}(r_{0},\theta_{0})$$

where $\eta_{m}^{n}(r_{0},\theta_{0})=J_{m}\left(\beta_{m}^{n}r_{0}\right)\cos(m\theta_{0})$ and $\Lambda_{\beta m}^{n}=\frac{1}{2}\left[{J_{m}}^{2}\left(\beta_{m}^{n}R\right)-J_{m-1}\left(\beta_{m}^{n}R\right)J_{m+1}\left(\beta_{m}^{n}R\right)\right]$.

Figure 1 represents the membrane drum geometrical characteristics.

### 2.1. Calculus of the Fluid Velocity Potential and Pressure over the Membrane

For a non-viscous fluid and with no cavitation, the motion induced by the vibration of the circular membrane in the fluid is described using the velocity potential; see Munson et al. [20]. This potential function $\varphi \left(r,\theta ,z,t\right)$ satisfies the wave equation for the fluid domain:

(10) $$\Delta \varphi -\frac{1}{c^{2}}\frac{\partial^{2}\varphi }{\partial t^{2}}=0$$

For harmonic motion $\varphi (r,\theta ,z,t)=\tilde{\varphi }(r,\theta ,z)\cdot e^{i\omega t}$, Helmholtz’s equation is deduced:

(11) $$\Delta \tilde{\varphi }+k^{2}\tilde{\varphi }=0$$

where k is the wave number, $k=\frac{\omega }{c}$, Δ the Laplacian operator, and c the sound speed related to the fluid bulk modulus B as $c=\frac{B}{\rho_{f}}$, and with fluid density $\rho_{f}$, together with the boundary conditions:

- For the wall boundaries, i.e., the cylindrical lateral and bottom circular surfaces of the cylindrical drum $\frac{\partial \varphi }{\partial n}=0$, with $\vec{n}$ in normal direction to the wall surfaces:

(12) $$\frac{\partial \varphi }{\partial z}=0\;z=0.\;\frac{\partial \varphi }{\partial r}=0\;r=R$$

- For the top membrane boundary, we obtain the following expression:

(13) $$\frac{\partial \varphi }{\partial z}=\frac{\partial \eta }{\partial t}\;\mathrm{for}\;z=H.\;0\leq r\leq R.\;0\leq \theta \leq 2\pi$$

Expanding in series considering the separation of variables in space–time coordinates, the velocity potential for the acoustic domain and the membrane deflection are expressed:

(14) $$\varphi (r,\theta ,z,t)=\sum_{m,n}\varphi_{m}^{n}(r,\theta ,z)\cdot \;f_{m}^{n}\left(t\right)$$

(15) $$\eta (r,\theta ,t)=\sum_{m,n}\eta_{m}^{n}(r,\theta )\cdot \;d_{m}^{n}\left(t\right)$$

where for harmonic motion

(16) $$f_{m}^{n}\left(t\right)={\bar{f}}_{m}^{n}\cdot e^{i\omega_{m}^{n}t}\;\mathrm{and}\;d_{m}^{n}\left(t\right)={\bar{d}}_{m}^{n}\cdot e^{i\omega_{m}^{n}t}$$

Considering Equation (10) for the potential, considering the boundary conditions (11), and applying separation of variables in spatial coordinates, the solution for the potential is as follows:

(17) $$\varphi (r,\theta ,z,t)=\sum_{m,n}\varphi_{m}^{n}\left(r,\theta \right)\cdot \cosh\left(\lambda_{m}^{n}z\right)\cdot \;f_{m}^{n}\left(t\right)$$

where the function

(18) $$\varphi_{m}^{n}\left(r,\theta \right)=J_{m}\left(\alpha_{m}^{n}r\right)\cos\left(m\theta \right)$$

where $\alpha_{m}^{n}$ comprises the roots of the equation $\frac{dJ_{m}\left(\alpha_{m}^{n}r\right)}{dr}=0,\;r=R$, i.e., the roots of equation

(19) $$J_{m-1}\left(\alpha_{m}^{n}R\right)-J_{m+1}\left(\alpha_{m}^{n}R\right)=0$$

and the parameter

(20) $$\lambda_{m}^{n}=\sqrt{{\left(\alpha_{m}^{n}\right)}^{2}-k^{2}}$$

Employing the boundary condition (13) of the fluid–membrane interface, the fluid velocity potential (17) and the membrane deflection (Equation (15)), and after integration over the membrane surface of the integrand squared series, the following expression is obtained:

(21) $$\begin{array}{l} {\int_{0}^{R}\int_{0}^{2\pi }\left[\sum_{m,n}\left(f_{m}^{n}\cdot \;J_{m}\left(\alpha_{m}^{n}r\right)\cos\left(m\theta \right)\;\cdot \lambda_{m}^{n}\cdot \sinh\left(\lambda_{m}^{n}H\right)\right)\right]}^{2}rdrd\theta =\\ ={\int_{0}^{R}\int_{0}^{2\pi }\left[\sum_{m,n}\left({\dot{d}}_{m}^{n}(t)\cdot \;J_{m}\left(\beta_{m}^{n}r\right)\cos\left(m\theta \right)\;\right)\right]}^{2}rdrd\theta \end{array}$$

From where it is deduced, taking into account the orthogonality of the harmonic functions and also the Bessel functions, considering the properties of the Lommel’s integral, the following expression is obtained; see Bowman [21]:

(22) $$f_{m}^{n}\left(t\right)=\frac{1}{\lambda_{m}^{n}\sinh\left(\lambda_{m}^{n}H\right)}\sqrt{\frac{\Lambda_{\beta m}^{n}}{\Lambda_{\alpha m}^{n}}}{\dot{d}}_{m}^{n}(t)$$

where $\Lambda_{\alpha m}^{n}=\frac{1}{2}\left[{J_{m}}^{2}\left(\alpha_{m}^{n}R\right)-J_{m-1}\left(\alpha_{m}^{n}R\right)J_{m+1}\left(\alpha_{m}^{n}R\right)\right]$ and $\Lambda_{\beta m}^{n}=\frac{1}{2}\left[{J_{m}}^{2}\left(\beta_{m}^{n}R\right)-J_{m-1}\left(\beta_{m}^{n}R\right)J_{m+1}\left(\beta_{m}^{n}R\right)\right]$.

The acoustic pressure in the fluid domain, neglecting gravity effects and for small displacements, is deduced from the momentum’s linearized equation; see Munson et al. [20]:

(23) $$\frac{\partial \varphi }{\partial t}+\frac{p}{\rho_{f}}=0$$

Considering Equation (17) for the potential and Equation (23), the acoustic pressure is deduced for the fluid domain:

(24) $$p(r,\theta ,z,t)=-\sum_{m,n}\frac{\rho_{f}\cosh\left(\lambda_{m}^{n}z\right)}{\lambda_{m}^{n}\sinh\left(\lambda_{m}^{n}H\right)}\sqrt{\frac{\Lambda_{\beta m}^{n}}{\Lambda_{\alpha m}^{n}}}\varphi_{m}^{n}\left(r,\theta \right){\ddot{d}}_{m}^{n}(t)$$

and the pressure over the membrane at z=H is

(25) $$p(r,\theta ,H,t)=-\sum_{m,n}\frac{\rho_{f}\coth\left(\lambda_{m}^{n}H\right)}{\lambda_{m}^{n}}\sqrt{\frac{\Lambda_{\beta m}^{n}}{\Lambda_{\alpha m}^{n}}}\varphi_{m}^{n}\left(r,\theta \right){\ddot{d}}_{m}^{n}(t)$$

Considering Equation (16) for harmonic motion, the following expression is obtained:

(26) $$p(r,\theta ,t)=\sum_{m,n}P_{m}^{n}(r,\theta )\;{\bar{d}}_{m}^{n}e^{i\omega_{m}^{n}t}$$

where $P_{m}^{n}$ is the pressure mode:

(27) $$P_{m}^{n}(r,\theta )=\;\frac{\rho_{f}\coth\left(\lambda_{m}^{n}H\right)}{\lambda_{m}^{n}}\sqrt{\frac{\Lambda_{\beta m}^{n}}{\Lambda_{\alpha m}^{n}}}\varphi_{m}^{n}\left(r,\theta \right){\omega_{m}^{n}}^{2}$$

### 2.2. Calculus of the Membrane Free Frequencies

The free frequencies of oscillation of the membrane are obtained by solving Equation (1) without the loading term. Keeping in mind Equations (5) and (25) for the membrane deflection and pressure over the membrane, Equation (1) is expressed in series of the associated modes as

(28) $$\sum_{m,n}T\Delta \eta_{m}^{n}\left(r,\theta \right)d_{m}^{n}(t)+\sum_{m,n}\rho_{m}t_{h}{\omega_{m}^{n}}^{2}\eta_{m}^{n}\left(r,\theta \right)d_{m}^{n}(t)=\sum_{m,n}P_{m}^{n}(r,\theta )d_{m}^{n}(t)$$

Taking into account Equation (27) for the pressure mode, the dynamic Equation (6) of the membrane in a vacuum, (5) for the membrane deflection, and (16) for harmonic motion, and after integration over the membrane surface of the integrand squared series of this equation, it can be deduced that

(29) $$\begin{array}{l} {\int_{0}^{R}\int_{0}^{2\pi }\left[\sum_{m,n}\left(-{\beta_{m}^{n}}^{2}T+\rho_{m}t_{h}{\omega_{m}^{n}}^{2}\right)\cdot J_{m}\left(\beta_{m}^{n}r\right)\cos\left(m\theta \right)d_{m}^{n}(t)\right]}^{2}rdrd\theta =\\ ={\int_{0}^{R}\int_{0}^{2\pi }\left[\sum_{m,n}\left(\frac{\rho_{f}\coth\left(\lambda_{m}^{n}H\right)}{\lambda_{m}^{n}}\sqrt{\frac{\Lambda_{\beta m}^{n}}{\Lambda_{\alpha m}^{n}}}{\omega_{m}^{n}}^{2}\;\right)\cdot J_{m}\left(\alpha_{m}^{n}r\right)\cos\left(m\theta \right)d_{m}^{n}(t)\right]}^{2}rdrd\theta \end{array}$$

Again, taking into account the orthogonality of the harmonic functions and also the Bessel functions, and considering the properties of Lommel’s integral (see Bowman [21]), the following analytic expression is obtained for the calculus of the associated mode frequencies:

(30) $$\Lambda_{\beta m}^{n}\left({\beta_{m}^{n}}^{4}T^{2}-2{\beta_{m}^{n}}^{2}T\rho_{m}t_{h}{\omega_{m}^{n}}^{2}+{\rho_{m}}^{2}{t_{h}}^{2}{\omega_{m}^{n}}^{4}\right)={\left[\frac{\rho_{f}\coth\left(\lambda_{m}^{n}H\right)}{\lambda_{m}^{n}}\sqrt{\frac{\Lambda_{\beta m}^{n}}{\Lambda_{\alpha m}^{n}}}\right]}^{2}{\omega_{m}^{n}}^{4}\Lambda_{\alpha m}^{n}$$

From here, the following quadratic equation is deduced:

(31) $$A_{m}^{n}\cdot {\omega_{m}^{n}}^{4}+B_{m}^{n}\cdot {\omega_{m}^{n}}^{\;2}+C_{m}^{n}=0$$

where

(32) $$A_{m}^{n}={\left[\frac{\rho_{f}\coth\left(\lambda_{m}^{n}H\right)}{\lambda_{m}^{n}}\right]}^{\;2}-{\left(\rho_{m}t_{h}\right)}^{2}$$

(33) $$B_{m}^{n}=2\rho_{m}\;t_{h}T\;{\beta_{m}^{n}}^{2}$$

(34) $$C_{m}^{n}=-T^{2}{\beta_{m}^{n}}^{4}$$

Because the parameter $\lambda_{m}^{n}$ in Equation (20) depends on the frequency wave number k, the solution is found by a fast convergent iteration procedure in an easy way.

### 2.3. Calculus of the Forced Frequency Response of the Membrane

The frequency spectrum response of the membrane is obtained by solving Equation (1) but now including the excitation load of frequency ω. Taking into account Equations (8), (15), and (26) for the membrane deflection, concentrated forcing load and acoustic pressure on the membrane, for harmonic motion, are expressed in a series of the associated modes as shown in Equation (1):

(35) $$\sum_{m,n}T\Delta \eta_{m}^{n}\left(r,\theta \right)d_{m}^{n}(t)+\sum_{m,n}\rho_{m}t_{h}\omega^{2}\eta_{m}^{n}\left(r,\theta \right)d_{m}^{n}(t)=\sum_{m,n}P_{m}^{n}(r,\theta )d_{m}^{n}(t)+\sum_{m,n}\eta_{m}^{n}\left(r,\theta \right)\;{\bar{q}}_{m}^{n}$$

Considering Equation (27) for the acoustic mode and (7) for the membrane deformation mode, and after integrating the squared series integrands of the membrane equation over the membrane surface, the following is deduced:

(36) $$\begin{array}{l} {\int_{0}^{R}\int_{0}^{2\pi }\left[\sum_{m,n}\left\{\left(-{\beta_{m}^{n}}^{2}T+\omega^{2}\rho_{m}t_{h}\right){\bar{d}}_{m}^{n}-{\bar{q}}_{m}^{n}\right\}\eta_{m}^{n}(r,\theta )\right]}^{2}rdrd\theta =\\ {\int_{0}^{R}\int_{0}^{2\pi }\left[\sum_{m,n}\left(\frac{\rho_{f}\coth\left(\lambda_{m}^{n}H\right)}{\lambda_{m}^{n}}\omega^{2}\sqrt{\frac{\Lambda_{\beta m}^{n}}{\Lambda_{\alpha m}^{n}}}\cdot \right)\varphi_{m}^{n}(r,\theta ){\bar{d}}_{m}^{n}\;\right]}^{2}\;rdrd\theta \end{array}$$

After substituting the deformation and potential modes, the following expression is obtained:

(37) $$\begin{array}{l} {\int_{0}^{R}\int_{0}^{2\pi }\left[\sum_{m,n}\left\{\left(-{\beta_{m}^{n}}^{2}T+\omega^{2}\rho_{m}t_{h}\right){\bar{d}}_{m}^{n}-{\bar{q}}_{m}^{n}\right\}J_{m}\left(\beta_{m}^{n}r\right)\cos\left(m\theta \right)\right]}^{2}rdrd\theta =\\ ={\int_{0}^{R}\int_{0}^{2\pi }\left[\sum_{m,n}\left(\frac{\rho_{f}\coth\left(\lambda_{m}^{n}H\right)}{\lambda_{m}^{n}}\omega^{2}\sqrt{\frac{\Lambda_{\beta m}^{n}}{\Lambda_{\alpha m}^{n}}}\cdot \right)J_{m}\left(\alpha_{m}^{n}r\right)\cos\left(m\theta \right){\bar{d}}_{m}^{n}\right]}^{2}\;rdrd\theta \end{array}$$

Keeping in mind the orthogonality of the modes $\eta_{m}^{n}$ and $\varphi_{m}^{n}$, a quadratic equation is obtained relating the deflection and loading coefficients ${\bar{d}}_{m}^{n}$ and ${\bar{q}}_{m}^{n}$:

(38) $$Y_{m}^{n}\cdot {\left(\frac{{\bar{d}}_{m}^{n}}{{\bar{q}}_{m}^{n}}\right)}^{2}+Z_{m}^{n}\cdot \left(\frac{{\bar{d}}_{m}^{n}}{{\bar{q}}_{m}^{n}}\right)+1=0$$

The relationship between the deflection coefficient and the forcing coefficient is calculated using a transfer function that depends on the frequency:

(39) $${\bar{d}}_{m}^{n}=TF\left(\omega \right)\cdot {\bar{q}}_{m}^{n}$$

where the transfer function is expressed as

(40) $$TF\left(\omega \right)=\frac{-Z_{m}^{n}\pm \sqrt{{\left(Z_{m}^{n}\right)}^{2}-4\cdot Y_{m}^{n}}}{2\cdot Y_{m}^{n}}$$

and the coefficients have the expressions

(41) $$Y_{m}^{n}=-\left[A_{m}^{n}\cdot \omega^{4}+B_{m}^{n}\cdot \omega^{2}+C_{m}^{n}\right]$$

(42) $$Z_{m}^{n}=\frac{B_{m}^{n}}{\rho_{m}\;t_{h}}-2\rho_{m}\;t_{h}\;\omega^{2}$$

## 3. Results

To validate the method, Table 1 compares the wet frequencies $f_{l\;m}^{n}$ (in Hz) of a membrane at the top position of a cylindrical cavity filled with water (an incompressible fluid) with the results of Tariverdilo et al. [22], who analysed the frequencies of the same membrane of an aluminium drum with a radius and height of 60 mm, and thickness of 0.5 mm. It shows small differences (relative discrepancy) between the results depending on the mode considered, and the calculated change in the free frequencies regarding the case in a vacuum $f_{v\;m}^{n}$.

The following graphical results are presented for the case of a membrane drum fully filled with a compressible fluid and the following reference characteristics: drum radius R=1 m, membrane thickness $t_{h}=0.02\;\mathrm{mm}$, drum height H=2 m, membrane material density $\rho_{m}=2700$$\;\frac{\mathrm{kg}}{m^{3}}$, fluid density $\rho_{f}=1.2250\;\frac{\mathrm{kg}}{m^{3}}$, and membrane tension $T=100\;\frac{N}{m}$.

We also present the excitation load $q_{0}$ of 1 N located at the reference point $r_{0}=0.20\;R$, $\theta_{0}=0.20{}^{\circ}$.

**Figure 2:**
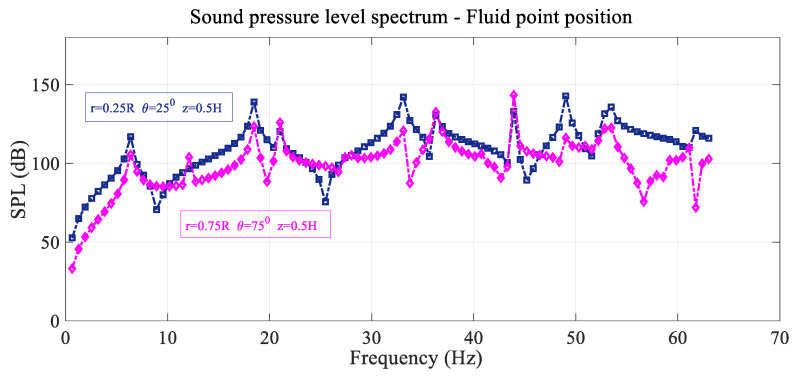
Variation in the sound pressure level SPL with frequency considering two different positions of the fluid.

The acoustic sound pressure level spectrums at two different interior points of the cylindrical cavity (first point is located at r=0.25 R, θ=25°, z=0.5 H and the second point is situated at r=0.75 R, θ=75°, z=0.5 H) are shown in Figure 2 in logarithmic scale as SPL (dB), with a forcing load $q_{0}$ of 1 N located at the reference point, with the SPL expressed as follows:

$$SPL\;(\mathrm{dB})=20\log\left(\frac{P}{P_{ref}}\right)\;\mathrm{with}\;P_{ref}=10^{-5}\;\mathrm{Pa}$$

In Figure 2, the sound pressure level spectrum depends on the situation of the fluid point inside the cavity. Resonant and antiresonant peaks appear in both cases, but the peak distribution is different for the second fluid point, and many of them coincide in frequency but not in amplitude. Some, but not all, of the different peaks, coincide in frequency with the free frequencies of the coupled system, but the number of peaks is slightly higher than the system-coupled free frequencies in the frequency range shown.

**Figure 3:**
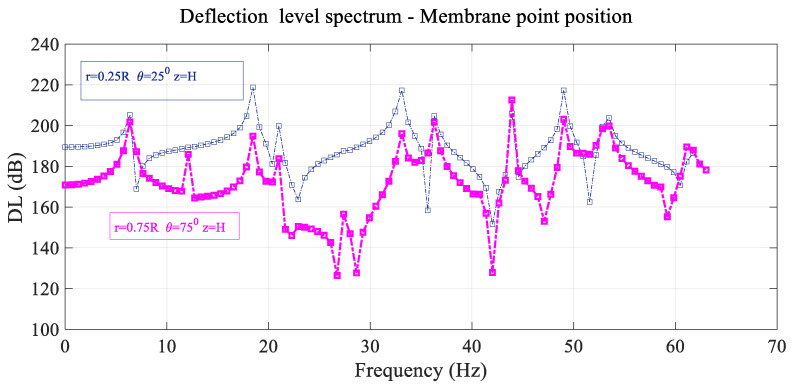
Variation in the membrane deflection level DL with frequency considering two different positions of the membrane.

The deflection level spectrums of two different points of the membrane (first point is placed at r=0.25 R θ=25° and the second point is situated at r=0.75 R θ=75°) are presented in Figure 3 in logarithmic scale as DL (dB), considering a forcing load $q_{0}$ of 1 N located at the reference point, with DL expressed as

$$DL(\mathrm{dB})=20\log\left(\frac{\eta }{\eta_{ref}}\right)\;\mathrm{with}\;\eta_{ref}=10^{-12}\;m$$

As in Figure 2, the deflection frequency spectrum of a point of the membrane depends on its location on the membrane, and many of the different resonant peaks have the same frequency value but different amplitude. This is not the case with the antiresonant peaks.

**Figure 4:**
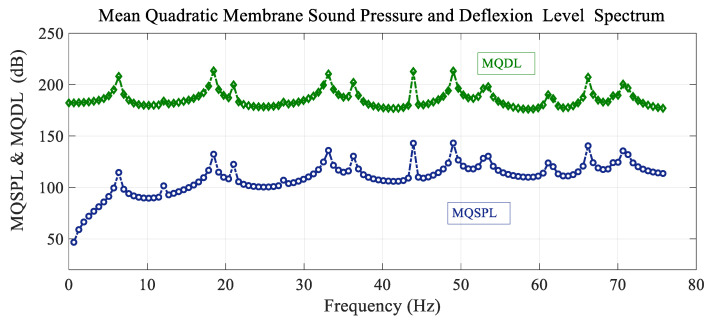
Variation in the mean quadratic sound pressure level and membrane deflection level MQSPL and MQDL with frequency.

Figure 4 shows the spectrum variation in the mean quadratic values of the sound pressure level and the deflection level of the membrane with the frequency (spectrum) of the forcing load situated at the reference point. There are a lot of peaks, and all of them coincide in frequency with the free frequencies of the system, but there are a few more natural frequencies in the range shown. The resonant peaks appear at the same frequency for both cases. The mean quadratic membrane sound pressure level is calculated as

$$MQSPL\;(\mathrm{dB})=20\log\left(\frac{MQP}{P_{ref}}\right)\;\mathrm{with}\;P_{ref}=10^{-5}\;\mathrm{Pa}$$

where MQP is the mean quadratic pressure over the membrane:

(43) $$MQP=\sqrt{\frac{1}{S_{m}}\iint_{S_{m}}{\left[\sum_{m,n}P_{m}^{n}(r,\theta )\;{\bar{d}}_{m}^{n}\right]}^{2}rd\theta }$$

and the mean quadratic membrane deflection level is expressed as

$$MQDL(\mathrm{dB})=20\log\left(\frac{MQD}{\eta_{ref}}\right)\;\mathrm{with}\;\eta_{ref}=10^{-12}\;m$$

where MQD is the mean quadratic deflection of the membrane:

(44) $$MQD=\sqrt{\frac{1}{S_{m}}\iint_{S_{m}}{\left[\sum_{m,n}\eta_{m}^{n}(r,\theta )\;{\bar{d}}_{m}^{n}\right]}^{2}rd\theta }$$

The resonant frequencies coincide for both the mean quadratic sound pressure and deflection levels.

**Figure 5:**
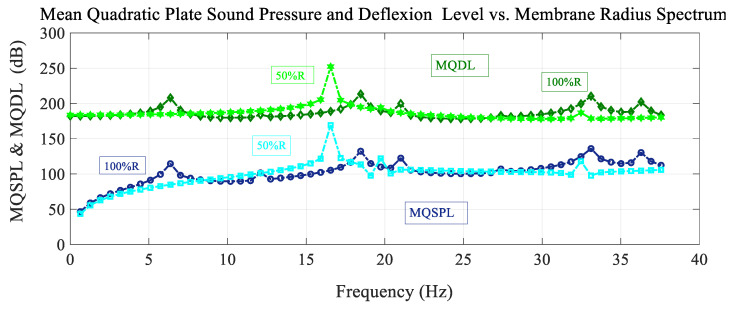
Variation in the mean quadratic sound pressure level and membrane deflection level MQSPL and MQDL with frequency for different values of the membrane radius.

Figure 5 shows the frequency spectrum of the mean quadratic values of the sound pressure level with the excitation load situated at the reference point, and also the deflection level of the membrane, for different values of the membrane radius. For the lower membrane radius, the peaks are shifted to higher values of the frequency. In the frequency range shown, there are more peaks for the reference case than for the case with a lower radius value. For the mean quadratic deflection level, antiresonant peaks also appear for lower values of the radius in the vicinity of 20 Hz.

**Figure 6:**
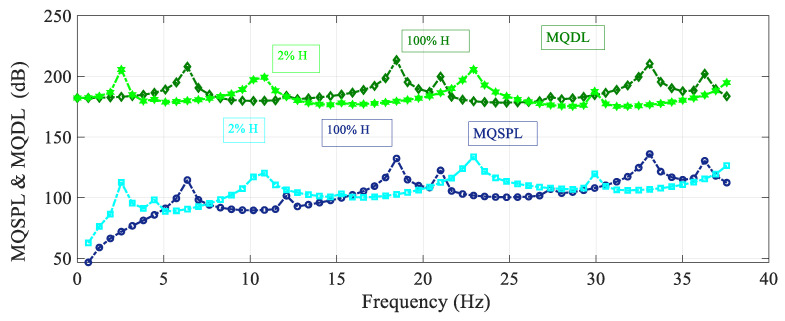
Variation in the mean quadratic sound pressure level and membrane deflection level MQSPL and MQDL with the frequency spectrum considering different values of container height.

Figure 6 shows the frequency spectrum of the mean quadratic values of the sound pressure level with the excitation load situated at the reference point, and also the deflection level of the membrane, for different values of container height. For the case of lower container height, the resonant peaks are shifted to lower values of the frequency. The peak frequencies are the same for the mean quadratic sound pressure and deflection levels. The peak amplitudes are also different for different heights, and there are no antiresonant peaks.

**Figure 7:**
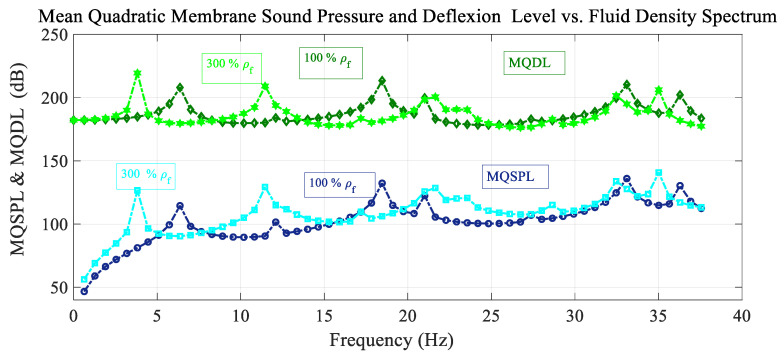
Variation in the mean quadratic sound pressure level and membrane deflection level MQSPL and MQDL with the frequency spectrum considering variation in fluid density.

Figure 7 shows the frequency spectrum of the mean quadratic values of the sound pressure level with the excitation load situated at the reference point and the deflection level of the membrane for different fluid density values. For the case of heavier fluid, the resonant peaks are shifted to lower values of the frequency, and the peak amplitudes are different. The amplitude of the peaks differs in both cases, and there are no antiresonant peaks.

**Figure 8:**
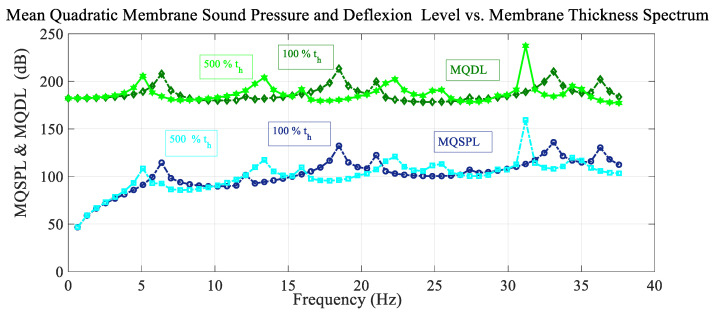
Variation in the mean quadratic sound pressure level and membrane deflection level MQSPL and MQDL with the frequency spectrum considering variation in membrane thickness.

Figure 8 shows the frequency spectrum of the mean quadratic values of the sound pressure level with the excitation load situated at the reference point, and also the deflection level of the membrane, for different values of membrane thickness. For cases of thicker membrane, the resonant peaks are shifted to lower values of the frequency. The amplitude of the peaks differs in both cases, with no antiresonant peaks.

**Figure 9:**
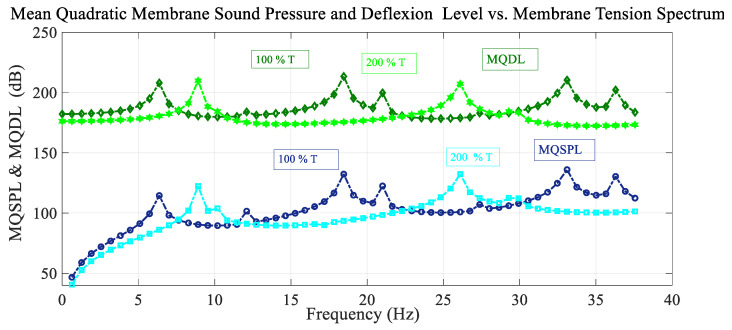
Variation in the mean quadratic sound pressure level and membrane deflection level MQSPL and MQDL with frequency considering variation in membrane tension.

Figure 9 shows the frequency spectrum of the mean quadratic values of the sound pressure level with the excitation load situated at the reference point and also the deflection level of the membrane, for different values of the membrane tension. For higher values of membrane tension, the peaks are shifted to higher values of frequency while presenting different amplitudes. The number of resonant peaks is smaller in the shown frequency range for the higher value of the membrane tension, and no antiresonant peaks appear.

**Figure 10:**
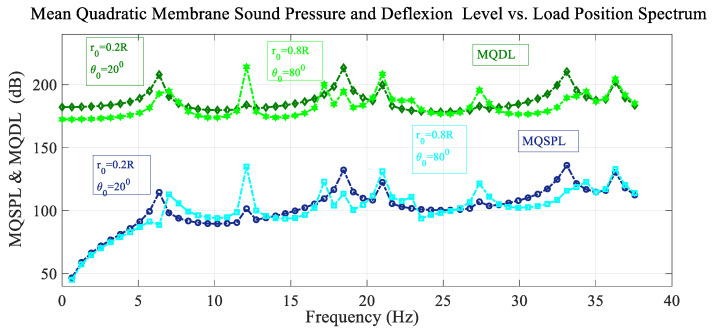
Variation in the mean quadratic sound pressure level and membrane deflection level MQSPL and MQDL with the frequency spectrum considering variation in load position.

Figure 10 shows the frequency spectrum of the mean quadratic values of the sound pressure level with the excitation load situated at the reference point, and also the deflection level of the membrane, for different values of load position. The peak configuration in amplitude and frequency is different; however, there are some peaks that coincide in frequency but have different amplitude. The two positions shown differ in both radial and azimuthal coordinates. For the new load position, the spectrum presents a few antiresonant peaks.

**Figure 11:**
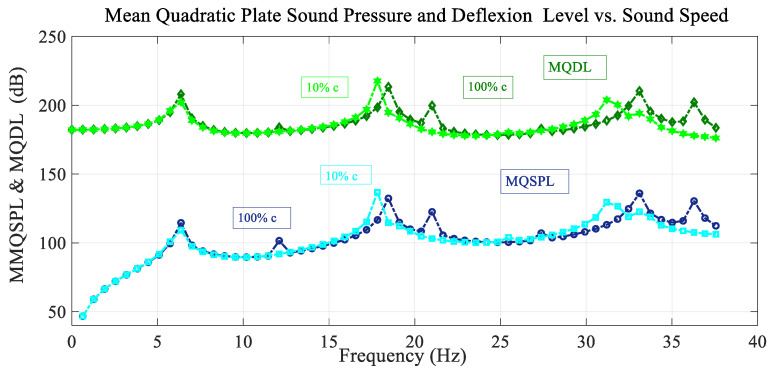
Variation in the mean quadratic sound pressure level and membrane deflection level MQSPL and MQDL with the frequency spectrum considering the variation in sound speed.

Figure 11 shows the frequency spectrum of the mean quadratic values of the sound pressure level with the excitation load situated at the reference point, and also the deflection level of the membrane, taking into account the compressibility effects or different sound speed values. The peak configuration is almost the same for frequencies lower than 10 Hz and is slightly shifted to the left and has different peak amplitude for higher values of frequency. There are fewer peaks for this higher frequency range. It must be said that, for an appreciable variation, in frequency spectrum with sound speed, it is necessary to take a value of sound speed with a great variation with respect to the reference case, as shown in the figure (10% vs. 100%). That is, for a small variation in sound speed, the change in spectrum is negligible with respect to the reference case.

**Figure 12:**
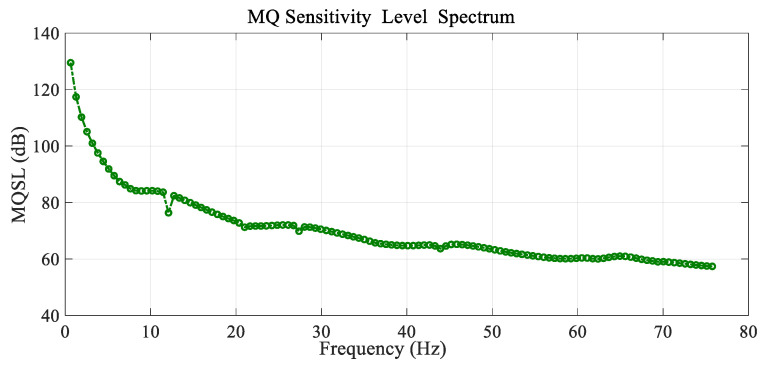
Variation in the mean quadratic sensibility level (MQSL) with the frequency spectrum for the reference conditions.

Figure 12 shows the variation in the mean quadratic sensitivity level (MQSL) with the frequency of the excitation load located at the reference point ($r_{0}=0.2\;R$ and $\theta_{0}=20{}^{\circ}$) and the reference conditions, or the mean quadratic sensitivity level spectrum for a frequency range of (0–80 Hz). This mean quadratic sensitivity level is calculated as

$$MQSL(\mathrm{dB})=20\log\left(\frac{MQS}{S_{ref}}\right)\;\mathrm{with}\;S_{ref}=10^{-7}\;\frac{m}{\mathrm{Pa}}$$

where MQS is the mean quadratic sensitivity of the membrane, defined as $MQS=\frac{MQD}{MQP}$.

The sensitivity decreases with the frequency of excitation and the rate of decrease is higher at lower values of the frequency spectrum in the range of 0–10 Hz. For higher values in the range of 10–70 Hz, the rate of decrease is lower and presents a slightly wavy variation, with some resonant and antiresonant peaks. This is an interesting result for the design of vibrating pressure sensors used in aeronautics, based on the vibration of a membrane or diaphragm in an air cavity, because high values of sensitivity are encountered at lower values of the frequency of excitation in these micro-electromechanical systems (MEMS).

**Figure 13:**
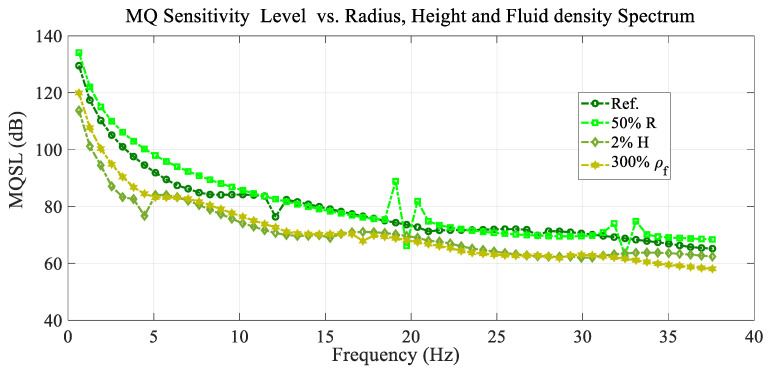
Variation in the mean quadratic sensibility level (MQSL) with the frequency spectrum for the reference conditions, and different values of the membrane radius, drum height, and fluid density.

Figure 13 shows the variation in the mean quadratic sensitivity level (MQSL) with the frequency or the mean quadratic sensitivity level spectrum for the excitation load located at the reference point ($r_{0}=0.2\;R$ and $\theta_{0}=20{}^{\circ}$) and the reference conditions, and for different values of membrane radius, drum height, and fluid density.

**Figure 14:**
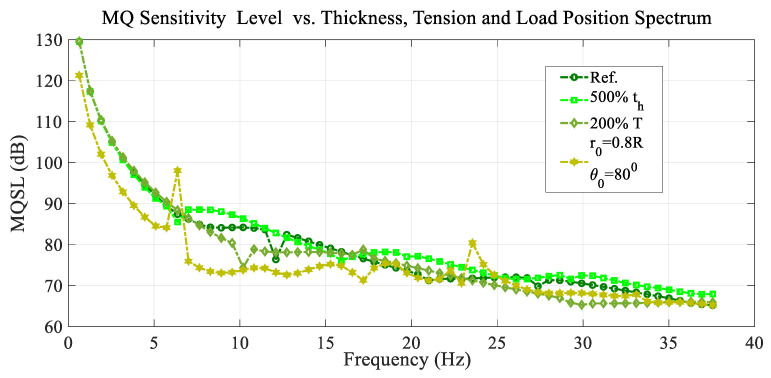
Variation in the mean quadratic sensibility level (MQSL) with the frequency spectrum for the reference conditions, and different values of the membrane thickness, membrane tension, and load position.

Figure 14 shows the variation in the mean quadratic sensitivity level (MQSL) with the frequency or the mean quadratic sensitivity level spectrum, for the excitation load located at the reference point ($r_{0}=0.2\;R$ and $\theta_{0}=20{}^{\circ}$) and the reference conditions, and for different values of the membrane thickness, membrane tension, and load position.

In general, all the curves in Figure 13 and Figure 14 present a decreasing trend with a high rate of decrease at lower frequency values, but there is a particular wavy variation in each one, including a few resonant and antiresonant peaks depending on the case considered.

Finally, Table 2 shows the frequencies of vibration in Hz of the membrane drum filled with incompressible fluid of air density for the different modes of the membrane, along with the values in a vacuum. The characteristics of the membrane correspond to the reference case. There are differences in the wet frequencies that are lower with respect to the vacuum case, depending on the mode considered.

## 4. Conclusions

Fluid structure interaction regarding a membrane at the top position of a cylindrical cavity fully filled with compressible inviscid fluid is analysed in the present work. Helmholtz’s wave equation is considered for the calculus of the fluid potential with the method of separation of variables, and the acoustic pressure is deduced considering Bernoulli’s linearized equation. By applying a mathematical integration procedure of the different terms expanded in series for the membrane equation while considering orthogonality, the coupled natural frequencies are calculated in a simple way using a quadratic equation with an iteration procedure, due to the dependence of the coefficients of this equation with the frequency. With this method, the results are analytically obtained in a simple and elegant manner. To validate the method, different results obtained by other authors show small discrepancies with those obtained in the present work. In the case of forced vibrations in an analogous manner after an integration procedure of the different terms of the dynamic equation considering the orthogonality properties regarding these integrals, the frequency response of the system is obtained by means of a transfer function. In particular, the deformation level spectrum of different points of the membrane and the sound level spectrum of different points inside the cavity are analysed. The mean quadratic level spectrums of the membrane sound pressure and deformation are also analysed, along with their variation with different parameters such as membrane radius, drum height, fluid density, membrane thickness, membrane tension, and load position. Moreover, the spectrum variation with the fluid compressibility (sound speed) is studied. Finally, the mean quadratic sensitivity level spectrum is analysed considering the variation with the mentioned parameters.

The results of the present modelling work can be applied in the design of a vibrating pressure sensor for use in aeronautics, or in the design of acoustic cabins of aircrafts, ships, and land vehicles where sound control is desired.

Finally, results are presented for the membrane of the cylindrical cavity filled with fluid with the density of air and a sound speed corresponding to the almost incompressible case in comparison with a vacuum.

## Figures and Tables

**Table 1:** Natural frequencies (Hz) for the membrane of the cylindrical drum of radius R=60 mm, drum height H=60 mm, and membrane thickness $t_{h}=0.5\;\mathrm{mm}$ (in a vacuum and water) in present method and the work of Tariverdilo et al. [22].

m, n	$f_{v}$	$f_{l}$ **(Tarverdilo)**	$f_{l}$ **(Present Method)**	**Relative** **Discrepancy (%)**
0, 0	54.9	--	15.4	
0, 1	46.5	--	46.5	
0, 2	85.3	--	85.3	
0, 3	123.3	--	123.3	
1, 0	87.5	19.2	17.0	11.4
1, 1	160.1	52.9	52.4	0.95
1, 2	232	94.6	93.2	1.48
1, 3	304	159	138.9	12.6
2, 0	117	32.7	29.6	2.77
2, 1	192	70.8	69.6	1.69
2, 2	265	115.7	113.5	1.90
2, 3	337	185.9	161.5	1.31
3, 0	145	46.7	43	7.92
3, 1	222	88.9	87	2.13
3, 2	297	136.8	134	2.05
3, 3	370	212.8	184	13.5
4, 0	173	61.2	57	6.86
4, 1	252	107.3	105	2.14
4, 2	328	157.9	155	1.84
4, 3	402	239.5	207	13.5

**Table 2:** Free frequencies (Hz) for a membrane made of aluminium of a cylindrical container fully filled with air and in a vacuum, considering different modes, with radius R=1 m, container height H=2 m, and membrane thickness $t_{h}=$ 0.02 mm.

m, n	$f_{v}$	$f_{a}$	**Frequency Reduction (%)**
0, 0	16.47	6.26	62.0
0, 1	37.81	18.37	51.4
0, 2	59.27	32.96	44.4
0, 3	80.76	49.10	39.2
1, 0	26.24	7.18	72.6
1, 1	48.05	20.94	56.4
1, 2	69.68	36.40	47.8
1, 3	91.26	53.19	41.7

## Data Availability

The original contributions presented in this study are included in the article. Further inquiries can be directed to the corresponding author.
